# The impact of Measles-Rubella vaccination on the morbidity and mortality from Congenital Rubella Syndrome in 92 countries

**DOI:** 10.1080/21645515.2018.1532257

**Published:** 2018-10-25

**Authors:** Emilia Vynnycky, Timoleon Papadopoulos, Konstantinos Angelis

**Affiliations:** a Modelling and Economics Unit, Public Health England, London, UK; b Centre for Mathematical Modelling of Infectious Diseases, Faculty of Epidemiology and Population Health, London School of Hygiene & Tropical Medicine, London, UK; c TB Modelling Group and TB Centre, London School of Hygiene & Tropical Medicine, London, UK

**Keywords:** GAVI, measles-rubella vaccination, campaigns, mathematical modelling, Congenital Rubella Syndrome

## Abstract

Since 2011, GAVI, The Vaccine Alliance, has funded eligible countries to introduce rubella-containing vaccination (RCV) into their national schedule. Two key indicators used to monitor the impact – the future deaths and DALYs (Disability Adjusted Life Years) averted through vaccination conducted in specific periods – are poorly understood for rubella and Congenital Rubella Syndrome (CRS). We calculate these indicators using an age-structured dynamic transmission model for rubella, with historical vaccination coverage projections during 2001–30 in 92 low and middle-income countries considered most likely to require global support to achieve the Global Vaccine Action Plan’s objectives. 131,000 CRS deaths and 12.5 million DALYs may be prevented with immunization campaigns at best-estimate coverage during 2001–30, relative to those without additional support. The impact depended on the time period considered and the method for attributing deaths averted to vaccination in specific periods. The analyses support ongoing activities to reduce CRS-related morbidity and mortality.

## Introduction

Approximately 105,000 children are born annually with Congenital Rubella Syndrome (CRS),^1^ a preventable cause of infant mortality, associated with lifelong disability, including cardiac defects, deafness, cataracts and mental retardation.^2^ Rubella vaccination is the primary method used to prevent CRS.^2^ The preferred strategy is to vaccinate a wide age-range (9 months to at least 15 years) in a campaign and then introduce routine infant rubella vaccination.^2^ Since 2012, GAVI, the Vaccine Alliance has funded eligible countries to conduct Measles-Rubella (MR) vaccination with this approach,^3,4^ which reduces rubella virus transmission in the population, and ensures that vaccinated girls are immune by child-bearing age.^2^ GAVI presently measures its progress in delivering strategic goals using the number of future deaths and Disability Adjusted Life Years (DALYs) averted through vaccination conducted in a given period with externally-supported vaccine.^5,6^

Although such indicators are helpful for contrasting the impact of vaccines for different diseases and vaccination in different periods, they are not straightforward to calculate and interpret for CRS. This follows from the facts that CRS-related disability and deaths are prevented many years after vaccination usually occurs, given that CRS may follow in a child if his/her non-immunised mother was infected with rubella when pregnant.^2^ When calculating the indicators, two factors then need to be accounted for when attributing disability and death averted due to vaccines administered during a given period. The first is whether a woman was vaccinated as a child. The second is the population-level immunity. This is influenced by the vaccination coverage in the population and it determines the amount of ongoing rubella transmission and therefore the risk of non-immunized mothers becoming infected when pregnant.^2^ Consequently, both the vaccination coverage among pregnant women during their childhood and the population-level coverage thereafter influence the disability and death averted due to vaccines administered in a specific period.

To date, no studies have either estimated the reduction in the burden of CRS that is attributable to vaccines administered in specific periods, accounting for these complications, or presented methods for calculating those reductions. Instead. modelling studies have considered the minimum level of coverage required to prevent increases in the burden of CRS^7^ and its sensitivity to the population birth rate and other factors,^8^ the impact of vaccination in the private sector on the burden of CRS,^9^ and the relative merits of introducing routine immunization compared to vaccinating teenage girls. This paper uses mathematical modelling to calculate the number of future deaths and DALYs averted until 2081 because of vaccination conducted in different periods during 2001–30, and contrasts different approaches for attributing the burden reduction to vaccination conducted in those periods. The estimates account for the long-term impact of vaccination and the amount of transmission when vaccinees reach adulthood.

## Results

### Deaths and DALYs averted

Table 1 summarises the estimated number of deaths and DALYs averted by each vaccination scenario, using different statistics for calculating the number of deaths among cases whose mothers would have been affected by vaccination in given periods. Supplementary Figures S.1 and S.2 show the annual and cumulative numbers of cases.

Using the base-case statistic, approximately 15, 75,000, 131,000, 41,000, 40,000 deaths were prevented with best-estimate SIA coverage alone compared to that without additional support, during 2001–10, 2001–20, 2001–30, 2011–15 and 2016–20 respectively (Table 1). These were similar to those calculated using statistics A and B, except for 2011–15 and 2016–20, for which they differed by approximately 50% and 25% respectively. Using statistic C led to increased predicted numbers of deaths because of SIAs conducted during 2001–20 and 2016–20. Compared to zero coverage, the deaths prevented by best-estimate SIA coverage alone ranged between 29,000 and 850,000 for 2001–10 and 2001–30 respectively, and the estimates obtained using different statistics generally differed by up to 20%.

Introducing routine vaccination without additional support during 2001–30 was predicted to prevent 9,000 additional deaths compared to SIAs alone conducted at best-estimate coverage, increasing to 60,000 if routine vaccination was conducted with best-estimate coverage. These estimates varied by vaccination period, decreasing to approximately 1000 and 4000 deaths respectively prevented when considering the period 2011–15, but were generally insensitive to the statistic used.

For each scenario and period, patterns in the number of DALYs prevented were similar to those for the number of deaths prevented. For the base-case statistic, best-estimate SIAs alone during 2001–30 were predicted to avert 12.5 million DALYs, compared to those without additional support, increasing to 80 million DALYs averted when comparing best-estimate SIAs against zero vaccination. For the same period, just under 1 million and 5 million DALYs were predicted to be averted through best-estimate coverage for both SIAs and routine vaccination, compared to best-estimate SIAs with routine vaccination without additional support or zero levels respectively. Considering 2011–15 and 2016–20, SIA vaccination alone at best-estimate coverage was predicted to prevent 4 million DALYs, compared to SIA vaccination alone conducted at the coverage expected without additional support.

### Sensitivity analyses

**Figure 1.**
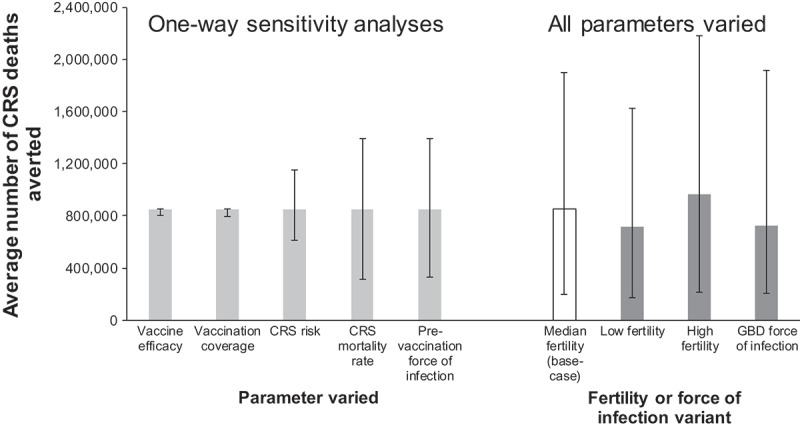
Sensitivity of estimates of the average number of CRS deaths prevented through best-estimate SIAs carried out during 2001–30, compared against no vaccination. The light grey bars show the values obtained for the base-case (median variant) fertility, with the thin bars reflecting the 95% range obtained after varying the parameter indicated on the x-axis individually. The thin bars on the dark grey or white bars show the 95% range obtained after varying all the parameters simultaneously, using either the median (base-case), low or high fertility or the pre-vaccination force of infection bootstrap datasets based on the Global Burden of Disease grouping for countries for which no seroprevalence datasets were available.

Comparing SIAs alone against no vaccination for 2001–30, the number of deaths prevented was relatively insensitive to the assumed variation in vaccine efficacy and coverage (Figure 1). The 95% range from varying the CRS risk following maternal infection was 600,000–1.2 million deaths prevented, increasing to 300,000–1.4 million deaths prevented when varying either the CRS mortality rate or pre-vaccination force of infection. When varying all parameters simultaneously, the 95% range became 182,000–1.8 million deaths prevented. Low and high fertility assumptions led to 20% lower and higher average numbers of deaths prevented respectively than those estimated for base-case parameter values (Figure 1). The estimated number and 95% range of deaths prevented resulting from basing the force of infection on GBD grouping for countries lacking seroprevalence data were similar to the base-case estimates.

**Figure 3.**
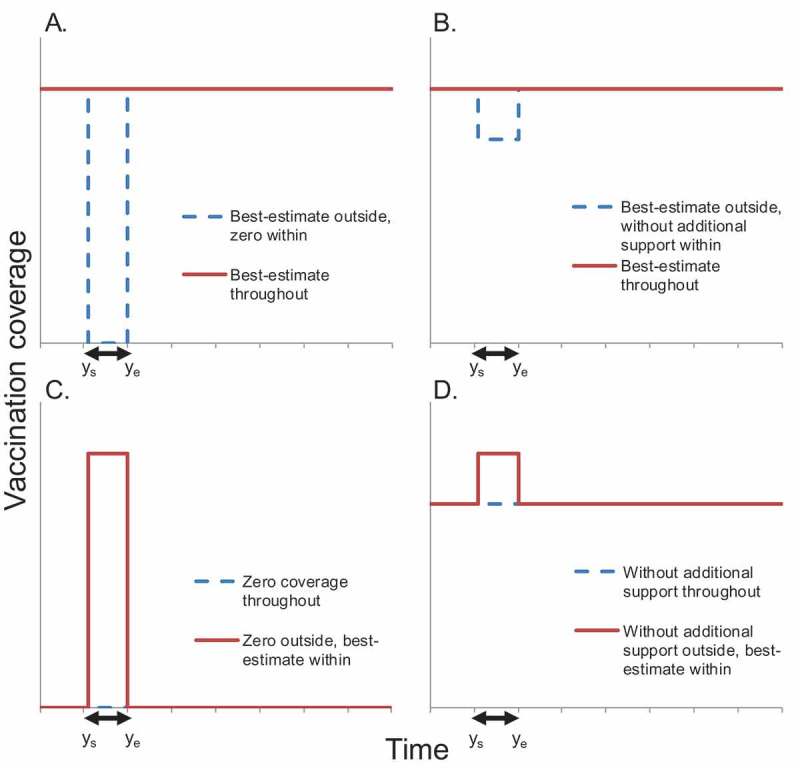
Schematic of the coverage used to calculate the number of deaths and DALYs averted from vaccination administered in a given period of interest (*y_s_-y_e_*), indicated by the double-headed arrow. Figures A and B show the two coverage assumptions used to estimate the impact of vaccination during a period of interest using the “best-estimate outside, reduced inside” approach. Figures C and D show the two coverage assumptions used to estimate the impact of vaccination using the “reduced outside, best-estimate inside” approach. For each scenario, the difference between the numbers of deaths associated with the period of interest with coverage set at that for the red line and that for the blue line gives the number of deaths averted. The numbers of deaths averted through best-estimate SIA vaccination conducted during 2011–15, for example, is calculated as the difference between the number of deaths among those born to mothers affected by vaccination during this period for the scenarios of no vaccination at all and zero coverage outside 2011–15 and best-estimate coverage during 2011–15.

**Figure 2.**
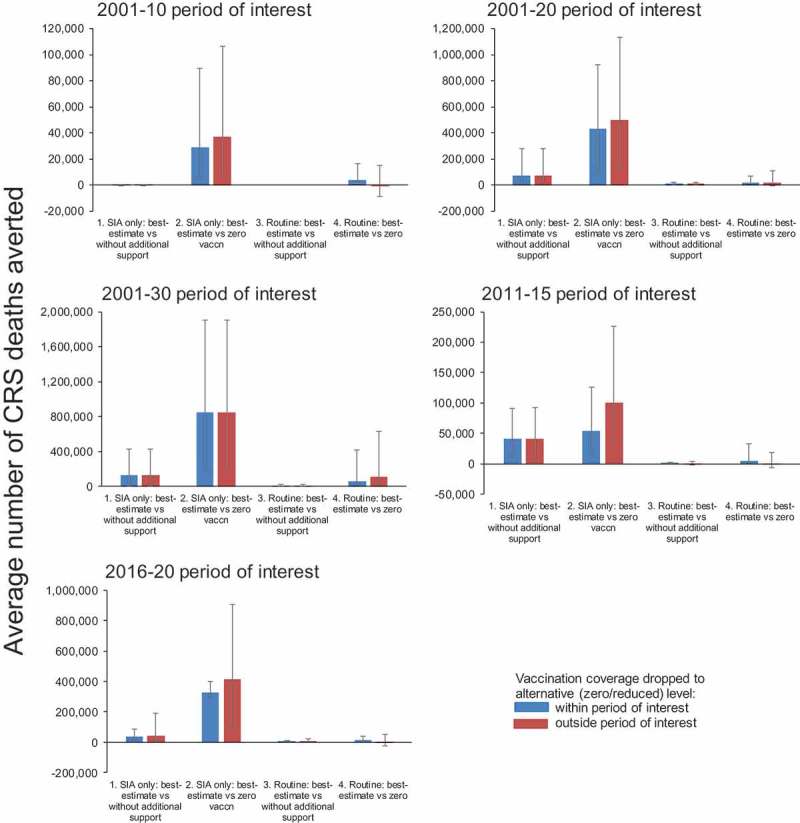
Summary of the average number of CRS deaths prevented through vaccination carried out during 2001–10, 2001–20, 2001–30, 2011–15 and 2016–20, calculated using the average number of deaths among people who would have affected by the vaccination carried out during the period of interest. The appropriate blue bars show the estimates obtained by keeping the vaccination coverage at best-estimate levels outside the period of interest at zero or levels without additional support during the period of interest; the red bars show the estimates obtained by keeping the vaccination coverage zero or at levels without additional support outside the period of interest, but increasing it to best-estimate coverage during the period of interest. The thin black bars show the 95% range obtained by varying all the model input parameters simultaneously.

When comparing best-estimate SIAs alone against zero vaccination, the “reduced outside, best-estimate inside” approach led to 20–100% higher estimated numbers of deaths prevented, than for the “best-estimate outside, reduced inside” approach for all periods except 2001–30 (Figure 2). It led to similar values for the other vaccination scenario comparisons, except when comparing best-estimate SIA and routine vaccination against best-estimate SIA coverage with no routine coverage, when increased numbers of deaths were predicted for several vaccination periods. For these, increasing the coverage to best-estimate levels from zero or that without additional support just for the vaccination period considered led to an increased predicted incidence and a negative predicted impact (Figures S.3 and S.4, Supplement).

## Discussion

We estimate that approximately 131,000 CRS deaths and 12.5 million DALYs may be prevented by increasing the coverage in SIAs from those expected without additional support to best-estimate levels in 92 countries during 2001–30, with 60,000 additional deaths and 5 million DALYs prevented by introducing routine vaccination. The morbidity and mortality prevented depended on the period considered, with approximately 40,000 deaths and 4 million DALYs prevented through SIAs conducted during 2011–15 and 2016–20. Approximately 850,000 CRS deaths and 80 million DALYs are predicted to be prevented through SIAs at best-estimate coverage, compared to zero vaccination.

Our analyses appear to be the first to estimate the reduction in the burden of CRS that may be attributable to vaccines administered in specific periods, also accounting for the complication that the outcome prevented (CRS) occurs many years after the vaccine is administered. As such, the reduction in the CRS burden that is attributable to vaccination in a given period is influenced both by the vaccination coverage among pregnant women during their childhood and the population-level coverage thereafter. Whilst our analyses focussed on rubella and CRS, analogous issues also apply to other infections for which the outcome prevented occurs many years after the vaccination is administered, such as hepatitis and HPV, for which vaccination may prevent liver and cervical cancers respectively. GAVI presently provides funding for eligible countries to introduce vaccines for both infections and so also measures its progress using the number of future deaths and DALYs averted through vaccination conducted in given periods for these infections.

We calculated the numbers of deaths among those born to mothers affected by vaccination in given periods using four statistics. The base-case and statistic A used the average number of deaths during given periods and statistics B and C used the total number of deaths since the period starts until 44 or 49 years after it finishes. The first two statistics have the advantage over the other two of being less sensitive to predictions of outbreaks. For example, statistic C predicted more deaths with best-estimate coverage for two periods than with coverage at levels which might be seen without additional support. This followed from predictions of many cases occurring towards the end of the period used in calculating the number of deaths, which outweighed the reduced number of deaths which had been predicted until then if the coverage was at levels expected without additional support during the periods of interest (Figure S.1, Supplement).

We used two approaches for estimating the impact of vaccination conducted during a period. The impact estimated from the “best-estimate outside, reduced inside” approach is interpretable as the contribution of vaccination conducted during that period to the impact of vaccination conducted from 2001 onwards. The “reduced outside, best-estimate inside” approach provides the literal definition of the impact of vaccination conducted during given periods, but has the disadvantage of comparing one scenario against one that could lead to increases in CRS incidence, such as best-estimate coverage within 2011–15 which decreases thereafter. This scenario reduces transmission during the vaccination period, leading to increases in the average age at infection for unvaccinated people, which, combined with increased transmission predicted once vaccination stops, leads to an increased CRS incidence and an apparently negative predicted impact of vaccination conducted during 2011–15.

Our analyses suggest that, considering the period 2001-10 very few deaths from CRS were prevented because of SIAs conducted at best-estimate coverage, compared to that with coverage which would have occurred without additional support. The reason for this low number is that the period 2001–10 predates the year when increased funding became available for countries to introduce Measles-Rubella vaccination. Consequently, for that period, the best-estimate coverage for SIAs is similar to the vaccination coverage which would have been seen without additional support.

Our analyses include several limitations. First, our estimates depend on the assumed pre-vaccination epidemiology of rubella, with datasets available for 30 of the 92 countries considered. These data, in turn, have several limitations,^1^ for example, being convenience samples from antenatal clinics, which may not represent the general population, and from cross-sectional surveys. For countries lacking serological data, data according to WHO or GBD region were used instead. We also note that several populous countries, including Afghanistan, Nigeria and Pakistan influence our estimated total number of CRS deaths prevented.

Second, for simplicity, we only included one dose of routine vaccination in our analyses, whereas two doses, including measles vaccine, are often provided. As we assumed that both the routine coverage was high and vaccine-derived immunity is lifelong, excluding the second dose would not have affected conclusions greatly: including it would just give the 5% of vaccinees without immunity after the first dose an opportunity to become immune.

Third, we may have overestimated the number of DALYS averted, as a country’s World Bank income group in 2017 determined their assigned DALY, with low-income groups assigned higher DALYS than high-income groups (29.2 vs 22.9 respectively). Such differences result from assumptions that high-income countries may provide better treatment for several CRS-related disabilities (e.g. cataract and deafness) than low-income countries.

A final limitation is that for simplicity, we did not account for the possibility that CRS cases may die many years after birth. The estimated CRS-related mortality rate to date has been based on short follow-up periods after birth (up to a year) and so may be an underestimate.

In conclusion, our analyses suggest that ongoing immunization activities could prevent substantial numbers of CRS-related deaths and DALYs. With increasing interest in measles elimination and introducing RCV, the number of deaths that are ultimately prevented through RCV may increase further. Further surveillance and serological studies are needed to improve the reliability of the estimated mortality prevented and monitor changes after introducing vaccination.

## Materials and methods

### Demographic data

We considered 92 low and middle-income countries (Table S.1, Supplement) which the Decade of Vaccines (DoV^10^ collaboration considered to be most likely to require global support to achieve the Global Vaccine Action Plan’s objectives.^11^ The following UN demographic country-specific data were used:^12^ a) Annual medium variant, sex-specific population size during 2001–2081, stratified by single-year age-groups; b) Age and sex-specific survival rates for 2010–15; c) Medium, high and low variants of the age-specific fertility rates in 5-year age groups projected until 2080; d) Crude birth rates for 2010–15.

### Description of the transmission model

#### General structure and demography

We used an age and sex-structured, deterministic, compartmental model of the transmission dynamics of rubella, following previous work.^1,13^ The population is stratified into those with maternal immunity (lasting 6 months), susceptible, pre-infectious (infected but not yet infectious), infectious and immune, using annual age bands and a “Realistic Age Structure.^14^ Country-specific birth and age-specific death rates were fixed at 2010–15 levels and calculated from UN population survival data for 2010–15 respectively.^12^ The supplement to^13^ provides the model’s differential equations.

#### The force of infection and pre-vaccination epidemiology of rubella

The force of infection (rate at which susceptibles are infected) changes over time and is calculated using the number of infectious individuals and the effective contact rate (rate at which infectious and susceptible individuals come into effective contact). Contact is described using the following matrix of “Who Acquires Infection From Whom”:

$$\left(\begin{array}{cc} \beta_{1} & 0.7\beta_{2}\\ 0.7\beta_{2} & \beta_{2} \end{array}\right)$$

The effective contact rate differs between < 13 and ≥ 13 year olds, with its relative size based on contact survey data.^15^ $\beta_{1}$ and $\beta_{2}$ are calculated from the average force of infection in < 13 and ≥ 13 year olds, estimated from age-stratified rubella seroprevalence data, which had been collected before RCV was introduced.^1^ Seroprevalence data were available for 25 countries as described in,^1^ with additional data (Supplement – sections A and B) for Cambodia,^16^ Democratic Republic of the Congo,^17^ Burkina Faso,^18^ Kenya^19^ and Tanzania^20^ identified through a systematic review, and unpublished data from Indonesia (*S Reef, personal communication, March 2015*). For countries lacking seroprevalence data, we used data from countries in the same WHO region (Supplement – section B and^1^). Confidence intervals (CI) on the force of infection were calculated using 1000 bootstrap-derived-seroprevalence datasets ^1^ and Supplement -section A).

#### Numbers of CRS cases, deaths and dalys

Country-specific numbers of CRS cases in year *y* during 2001–2080 were calculated by summing the number of CRS cases born each day to women aged 15–44 years (Supplement – section C). As assumed elsewhere,^1,9,13^ infection during the first 16 weeks of pregnancy carries a 65% risk of the newborn having CRS (Table 2). The number of CRS deaths in year *y* was calculated by multiplying the number of CRS cases born in year *y* by the assumed case fatality rate (30% – see Table 2). The number of DALYs for cases in year *y* was calculated by multiplying the number of CRS cases in year *y* by the corresponding DALY (from^29^), which was based on the country-specific World Bank Income group for 2017.^30^ Both the DALYs and the assigned World Bank income group remained fixed over time.

**Table 2.** Summary of the basecase and ranges of the parameters used in the model.

	Base-case value	Values used in sensitivity analyses	Basis
Pre-vaccination force of infection (used to calculate contact parameters)	Based on pre-vaccination seroprevalence data from the country (if available) or from the same WHO region otherwise.	1000 bootstrap-derived values	See ^1^.
Vaccine efficacy	95%	85% to 99%, sampled from the truncated Beta distribution with parameters α = 33 and ß = 2.	Plausible values
CRS-related mortality rate	30%	Sampled from the uniform distribution in the range 10–50%.	3 studies in Vietnam, Greece and Panama in which the 95% confidence intervals were 20–51%, 12–50% and 15–40% respectively ^21–23^.
Vaccination coverage	From historical projections^24^	10% higher or lower each year than historical projections.	Plausible
Risk of a child being born with CRS if the mother is infected during the first 16 weeks of pregnancy	65%	Sampled from the Gamma distribution with shape and scale parameters 37 and 56 respectively.	Lead to a median and 95% range of 65% and 47–88% respectively consistent with those from several studies^25-27^ which, as found in a recent review^28^ were likely to have been more reliable than those in other studies.

**Table 1.** Estimates of the average number of CRS deaths and DALYS prevented through SIAs, with or without routine RCV vaccination carried out during 2001–10, 2001–20, 2001–30, 2011–15 and 2016–20 using different statistics for the number of cases among mothers affected by vaccination during a given period. See the main text for a description of the statistics.

		Deaths averted					DALYs averted				
Comparison	Statistic	2001–10	2001–20	2001–30	2011–15	2016–20	2001–10	2001–20	2001–30	2011–15	2016–20
1. Best-estimate SIA alone vs SIA without additional support alone	Base-case	15	74,728	130,701	40,772	39,523	1362	7,139,512	12,509,331	3,851,097	3,798,699
	A.	19	70,721	116,217	33,491	40,098	1733	6,725,936	11,087,188	3,161,694	3,837,194
	B.	94	63,490	189,638	87,951	55,946	8669	5,816,900	18,261,846	8,387,707	5,257,221
	C	26	−45,299	108,614	6554	−27,674	2427	−4,285,580	10,626,905	467,943	−2,595,230
2. Best-estimate SIA alone vs no vaccination	Base-case	29,223	430,497	851,435	53,655	328,790	2,584,411	40,043,759	79,877,605	5,040,269	30,653,439
	A.	26,920	375,495	805,343	45,893	273,475	2,382,642	34,891,178	75,501,986	4,306,514	25,502,913
	B.	22,381	436,325	779,153	94,624	322,712	1,987,139	40,262,952	72,874,068	9,001,766	29,761,100
	C	22,527	351,463	649,738	60,410	256,315	2,000,410	32,358,531	60,782,296	5,455,182	23,645,429
3. Best-estimate routine and SIA vs best estimate SIA and routine vaccination without additional support	Base-case	0	8837	8724	1167	9358	0	825,997	814,591	108,333	873,833
	A.	0	8905	8994	1136	9383	0	832,654	840,880	105,423	876,527
	B.	0	8809	8704	1167	9330	0	823,264	812,602	108,333	871,100
	C	0	8806	8704	1167	9327	0	823,057	812,602	108,333	870,893
4. Best-estimate routine vaccination and SIA vs best-estimate SIA and no routine vaccination	Base-case	3984	18,498	57,912	4107	11,946	359,440	1,705,313	4,957,446	367,880	1,107,205
	A.	3813	18,236	52,741	3761	11,782	344,760	1,682,343	4,542,776	339,174	1,093,868
	B.	4488	19,373	58,198	4700	12,861	405,581	1,785,642	4,982,807	421,517	1,187,713
	C	4570	19,095	58,198	4686	12,507	413,122	1,759,933	4,982,816	420,312	1,155,045

### Deaths and dalys averted

#### Vaccination coverage definitions and scenarios

In these analyses, we define the “best-estimate coverage” as the highest realistic vaccination coverage which might be attained in a country and the “Coverage without additional support” as the coverage that might be seen if a country receives no further external support. In practice, a country may attain best-estimate coverage if it receives additional external support. By definition, the best estimate coverage equals the coverage seen without additional support in countries which introduced RCV without having received additional external support.

We calculated the average number of CRS deaths and DALYs prevented by vaccination conducted during 2001–2010, 2001–2020, 2001–2030, 2011–2015, 2016–2030 for the following:

1. Special Immunization Activities (SIAs) at best-estimate coverage compared to SIAs conducted without additional support, both without routine immunization;
2. SIAs at best-estimate coverage, without routine vaccination compared to no vaccination;
3. Both routine and SIA vaccination at best-estimate coverage, compared to SIAs at best-estimate coverage without routine vaccination;
4. Both routine and SIA vaccination at best-estimate coverage compared to routine vaccination without additional support but with SIAs at best-estimate coverage.

The projected vaccination coverage was based on Gavi’s Strategic Demand Forecast, version 1^24^ and the historical coverage during SIAs and routine vaccination came from WHO and WUENIC estimates for measles-containing vaccine (MCV1) respectively.^31,32^ To facilitate between-scenario comparisons, 2000 was the earliest year for introducing vaccination.

For simplicity, routine vaccination is provided as a single dose in the model. Comparisons 1 and 2 demonstrate the incremental impact of best-estimate coverage in campaigns (relative to that without additional support or no vaccination), and include hypothetical scenarios, as they consider campaigns in the absence of routine immunization. In reality, the latter would be necessary for introducing rubella vaccination. Comparisons 3 and 4 show the incremental effect of adding routine vaccination to vaccination in mass campaigns.

#### Attributing deaths and DALYs prevented to vaccination conducted in specific periods

In the base-case for each comparison we used a “best-estimate outside, reduced inside” approach (Figure 3A,B) to calculate the numbers of deaths and DALYs averted by vaccination administered during the period $y_{s}-y_{e}$, where $y_{s}$ and $y_{e}$ are the first and last years of the period. Considering deaths for comparisons 1 and 2, this number was calculated as the difference in the number of CRS deaths associated with the period (see definitions below) with SIAs at best-estimate coverage and the corresponding number of deaths for the same scenario but with SIA coverage at the alternative (reduced) level within the period ($y_{s}-y_{e}$). The calculation for comparisons 3 and 4 and DALYs is analogous.

We define the number of CRS deaths that are associated with a period $y_{s}-y_{e}$, (denoted $G_{y_{s}y_{e}}^{C}$) as the number of CRS deaths among those CRS cases whose mothers would have been affected by vaccination conducted during $y_{s}-y_{e}$. For a given coverage, *c*, during $y_{s}-y_{e}$, this was calculated as the average of the cumulative number of CRS deaths from the start of the period until 14–49 years after the period ends, as follows:

$$G_{y_{s}y_{e}}^{c}=\overset{49}{\underset{i=14}{\sum }}\frac{Dy_{s},y_{e}+i}{36}$$

where $Dy_{s},y_{e}+i$ is the total number of CRS deaths from years $y_{s}$ to $y_{e}+i$. The summation covers the reproductive lifespan of people vaccinated during $y_{s}-y_{e}$. The number of deaths and DALYs averted were summed for all countries.

### Sensitivity analyses

We also estimated the numbers of deaths (and similarly, DALYS) prevented by vaccination conducted in the periods of interest using alternative statistics for the number of deaths among cases whose mothers would have been affected by vaccination administered during $y_{s}-y_{e}$:

A. The average of the cumulative number of CRS deaths since the period starts (y_s_) until 49 years from its end $\left(\overset{49}{\underset{i=0}{\sum }}\frac{Dy_{s},y_{e}+i}{50}\right)$.
B. The total number of deaths since the period starts until 44 years from its end $\left(D_{y_{s,}y_{e}+44}\right)$.
C. The total number of deaths since the period starts until 49 years from its end $\left(D_{y_{s,}y_{e}+49}\right)$.

We estimated the sensitivity of the base-case impact statistic to the input parameters by calculating the 95% range of its values after sampling each parameter in Table 2 1000 times individually and simultaneously. Point estimates and the 95% range of the outcomes were also calculated using:

1. UN population projections of high and low variants of the fertility rates.
2. Bootstrap-derived values for the force of infection compiled from seroprevalence data from countries in the same Global Burden of Disease (GBD) region instead of the same WHO regio^33^ (Table S.5, Supplement) for countries which had no seroprevalence data.

Finally, we explored the effect of the “reduced outside, best-estimate within” approach (Figure 3C,D) on the estimated number of deaths averted, i.e. using vaccination at zero/reduced coverage outside the period considered and best-estimate levels within it, using the base-case statistic to calculate the number of deaths among cases whose mothers were affected by vaccination during the period.

## Abbreviations

CRS: Congenital Rubella Syndrome
DALY: Disability Adjusted Life Years
MR: Measles-Rubella
RCV: rubella-containing vaccine
