# Supplementary material for: The impact of Measles-Rubella vaccination on the morbidity and mortality from Congenital Rubella Syndrome in 92 countries
Source: Hum Vaccin Immunother. 2018 Oct 25;15(2):309–16. doi: 10.1080/21645515.2018.1532257 (PMC6422479; doi:10.1080/21645515.2018.1532257)
Supplement: Supplemental Material [file khvi-15-02-1532257-s001.docx]

**The impact of Measles-Rubella vaccination on the morbidity and mortality from Congenital Rubella Syndrome in 92 countries**

**Supplementary Material**

# A: Countries analysed and sources of the bootstrap datasets

Confidence intervals (CI) on the force of infection estimated from each seroprevalence dataset were generated using 1000 bootstrap-derived seroprevalence datasets, as described in (1). Briefly, for countries with multiple seroprevalence datasets, CI were defined using 1000 bootstrap-derived-seroprevalence datasets compiled using equal numbers of bootstrap-derived values from each original dataset, or proportionately to the urban and rural population size, where possible. If no datasets were available, we defined the range using all bootstrap-derived values from the same WHO region (Section B, Supplement). If the force of infection was reproduced using multiple datasets, the point estimate was calculated using contact parameters associated with the median from 1000 bootstrap samples of the pre-vaccination unweighted CRS incidence/100,000 livebirths among 15-44 year olds.

Table S.1: Summary of the countries analysed and the bootstrap datasets used to define the pre-vaccination force of infection for each country, using either the WHO regional or GBD grouping to assign datasets for countries without serological datasets from before the introduction of RCV. See Table S.2 and Table S.3 for the datasets used to make up the bootstrap datasets. See (1), (64) and Table S.4, for the best-fitting estimates of the pre-vaccination force of infection and CRS incidence for each dataset.

| **Country** | **World Bank income group, 2017** | **DALY (GBD 2010 disability weights)** | **Seroprevalence data used to generate the bootstrap samples, based on:** | |
| --- | --- | --- | --- | --- |
|  |  |  | **WHO regional grouping** | **GBD regional grouping** |
| Afghanistan | Low income | 29.2 | EMRO region | Asia, South |
| Albania | Upper middle income | 22.9 | EURO region | Europe, Central |
| Angola | Lower middle income | 27.8 | AFRO region | Sub-Saharan Africa, Central |
| Armenia | Lower middle income | 27.8 | EURO region | Asia, Central |
| Azerbaijan | Upper middle income | 22.9 | EURO region | Asia, Central |
| Bangladesh | Lower middle income | 27.8 | Bangladesh, 2004-5(56) | Bangladesh, 2004-5(56) |
| Belize | Upper middle income | 22.9 | Caribbean | Caribbean |
| Benin | Low income | 29.2 | Benin, 1993(2) | Benin, 1993(2) |
| Bhutan | Lower middle income | 27.8 | SEARO region | Asia, South |
| Bolivia | Lower middle income | 27.8 | AMRO region, excluding the Caribbean | Latin America, Andean |
| Bosnia and Herzegovina | Upper middle income | 22.9 | EURO region | Europe, Central |
| Burkina Faso | Low income | 29.2 | Burkina Faso, 2007-8(3) | Burkina Faso, 2007-8(3) |
| Burundi | Low income | 29.2 | AFRO region | Sub-Saharan Africa, East |
| Cambodia | Lower middle income | 27.8 | Cambodia, 2012(64) | Cambodia, 2012(64) |
| Cameroon | Lower middle income | 27.8 | AFRO region | Sub-Saharan Africa, West |
| Cape Verde | Lower middle income | 27.8 | AFRO region | Sub-Saharan Africa, West |

| **Country** | **World Bank income group, 2017** | **DALY (GBD 2010 disability weights)** | **Seroprevalence data used to generate the bootstrap samples, based on:** | |
| --- | --- | --- | --- | --- |
|  |  |  | **WHO regional grouping** | **GBD regional grouping** |
| Central African Republic | Low income | 29.2 | AFRO region | Sub-Saharan Africa, Central |
| Chad | Low income | 29.2 | AFRO region | Sub-Saharan Africa, West |
| Comoros | Low income | 29.2 | AFRO region | Sub-Saharan Africa, East |
| Congo, Democratic Republic | Low income | 29.2 | Democratic Republic of the Congo (Kikwit, Mikalayi, Tshikapa, Vanga), 2008-9(7) | Democratic Republic of the Congo (Kikwit, Mikalayi, Tshikapa, Vanga), 2008-9(7) |
| Congo, Republic of | Lower middle income | 27.8 | Congo, <1991(4); | Congo, <1991(4); |
| Cote d'Ivoire | Lower middle income | 27.8 | Cote d'Ivoire, 1975(5) & 1985-6(6) | Cote d'Ivoire, 1975(5) & 1985-6(6) |
| Cuba | Upper middle income | 22.9 | Caribbean | Caribbean |
| Djibouti | Lower middle income | 27.8 | EMRO region | Sub-Saharan Africa, East |
| Egypt | Lower middle income | 27.8 | EMRO region | North Africa / Middle East |
| El Salvador | Lower middle income | 27.8 | AMRO region, excluding the Caribbean | Latin America, Central |
| Eritrea | Low income | 29.2 | AFRO region | Sub-Saharan Africa, East |
| Ethiopia | Low income | 29.2 | Ethiopia, 1981(8) & 1994(9) | Ethiopia, 1981(8) & 1994(9) |
| Fiji | Upper middle income | 22.9 | Fiji, <1973(65) | Fiji, <1973(65) |
| Gambia | Low income | 29.2 | AFRO region | Sub-Saharan Africa, West |
| Georgia | Lower middle income | 27.8 | EURO region | Asia, Central |
| Ghana | Lower middle income | 27.8 | Ghana, 1997(11) | Ghana, 1997(11) |
| Guatemala | Lower middle income | 27.8 | AMRO region, excluding the Caribbean | Latin America, Central |
| Guinea | Low income | 29.2 | AFRO region | Sub-Saharan Africa, West |
| Guinea-Bissau | Low income | 29.2 | AFRO region | Sub-Saharan Africa, West |
| Guyana | Upper middle income | 22.9 | Caribbean | Caribbean |

| **Country** | **World Bank income group, 2017** | **DALY (GBD 2010 disability weights)** | **Seroprevalence data used to generate the bootstrap samples, based on:** | |
| --- | --- | --- | --- | --- |
|  |  |  | **WHO regional grouping** | **GBD regional grouping** |
| Haiti | Low income | 29.2 | Haiti, 2003(32) | Haiti, 2003(32) |
| Honduras | Lower middle income | 27.8 | AMRO region, excluding the Caribbean | Latin America, Central |
| India | Lower middle income | 27.8 | India, 1968 (urban & rural Delhi)(57), 1972-3 (Chandrigarh & Lucknow)(57), 1976 (Calcutta)(58), <1987 (Delhi)(59), <1990 (Delhi)(60), 1999-2000 (urban and rural Vellore)(61) | India, 1968 (urban & rural Delhi)(57), 1972-3 (Chandrigarh & Lucknow)(57), 1976 (Calcutta)(58), <1987 (Delhi)(59), <1990 (Delhi)(60), 1999-2000 (urban and rural Vellore)(61) |
| Indonesia | Lower middle income | 27.8 | Indonesia, 2007 (*S Reef, personal communication, March 2015*) | Indonesia, 2007 (*S Reef, personal communication, March 2015*)) |
| Iraq | Upper middle income | 22.9 | EMRO region | North Africa / Middle East |
| Kenya | Lower middle income | 27.8 | Kenya, 1996-9 (Kilifi)(12, 13) and 2005 (Eldoret) (14); | Kenya, 1996-9 (Kilifi)(12, 13) and 2005 (Eldoret) (14); |
| Kiribati | Lower middle income | 27.8 | WPRO, excluding China & Australia | Oceania |
| Korea, Democratic People’s Republic | Low income | 29.2 | WPRO region, excluding China & Australia | Asia Pacific, high income |
| Kyrgyzstan | Lower middle income | 27.8 | Kyrgyzstan, 2001(51) | Kyrgyzstan, 2001(51) |
| Lao, People’s Democratic Republic | Lower middle income | 27.8 | WPRO region, excluding China & Australia | Asia, Southeast |
| Lesotho | Lower middle income | 27.8 | AFRO region | Sub-Saharan Africa, Southern |
| Liberia | Low income | 29.2 | AFRO region | Sub-Saharan Africa, West |
| Madagascar | Low income | 29.2 | Madagascar, 1990-1995 (15) | Madagascar, 1990-1995 (15) |
| Malawi | Low income | 29.2 | AFRO region | Sub-Saharan Africa, East |
| Mali | Low income | 29.2 | AFRO region | Sub-Saharan Africa, West |

| **Country** | **World Bank income group, 2017** | **DALY (GBD 2010 disability weights)** | **Seroprevalence data used to generate the bootstrap samples, based on:** | |
| --- | --- | --- | --- | --- |
|  |  |  | **WHO regional grouping** | **GBD regional grouping** |
| Mauritania | Lower middle income | 27.8 | AFRO region | Sub-Saharan Africa, West |
| Micronesia | Lower middle income | 27.8 | WPRO region, excluding China & Australia | Oceania |
| Moldova | Lower middle income | 27.8 | EURO region | Europe, Eastern |
| Mongolia | Lower middle income | 27.8 | WPRO region, excluding China & Australia | Asia, Central |
| Morocco | Lower middle income | 27.8 | Morocco, 1969-1970(39) | Morocco, 1969-1970(39) |
| Mozambique | Low income | 29.2 | Mozambique, 2002(16) | Mozambique, 2002(16) |
| Myanmar | Lower middle income | 27.8 | SEARO region | Asia, Southeast |
| Nepal | Low income | 29.2 | Nepal, 2008(62) | Nepal, 2008(62) |
| Nicaragua | Lower middle income | 27.8 | AMRO region, excluding the Caribbean | Latin America, Central |
| Niger | Low income | 29.2 | AFRO region | Sub-Saharan Africa, West |
| Nigeria | Lower middle income | 27.8 | AFRO region | Sub-Saharan Africa, West |
| Pakistan | Lower middle income | 27.8 | Pakistan, <1997(40) & 1999-2004(41) | Pakistan, <1997(40) & 1999-2004(41) |
| Papua New Guinea | Lower middle income | 27.8 | WPRO region, excluding China & Australia | Oceania |
| Paraguay | Upper middle income | 22.9 | AMRO region, excluding the Caribbean | Latin America, Tropical |
| Philippines | Lower middle income | 27.8 | WPRO region, excluding China & Australia | Asia, Southeast |
| Rwanda | Low income | 29.2 | AFRO region | Sub-Saharan Africa, East |
| Samoa | Upper middle income | 22.9 | WPRO region, excluding China & Australia | Oceania |
| Sao Tome e Principe | Lower middle income | 27.8 | AFRO region | Sub-Saharan Africa, West |
| Senegal | Low income | 29.2 | Senegal, 1996-2001 (20) | Senegal, 1996-2001 (20) |
| Sierra Leone | Low income | 29.2 | AFRO region | Sub-Saharan Africa, West |

| **Country** | **World Bank income group, 2017** | **DALY (GBD 2010 disability weights)** | **Seroprevalence data used to generate the bootstrap samples, based on:** | |
| --- | --- | --- | --- | --- |
|  |  |  | **WHO regional grouping** | **GBD regional grouping** |
| Solomon Islands | Lower middle income | 27.8 | WPRO region, excluding China & Australia | Oceania |
| Somalia | Low income | 29.2 | EMRO region | Sub-Saharan Africa, East |
| Sri Lanka | Lower middle income | 27.8 | SEARO region | Asia, Southeast |
| Sudan, North | Lower middle income | 27.8 | EMRO region | Sub-Saharan Africa, East |
| Sudan, South | Low income | 29.2 | EMRO region | Sub-Saharan Africa, East |
| Swaziland | Lower middle income | 27.8 | AFRO region | Sub-Saharan Africa, Southern |
| Syria | Lower middle income | 27.8 | EMRO region | North Africa/ Middle East |
| Tajikistan | Lower middle income | 27.8 | EURO region | Asia, Central |
| Tanzania | Low income | 29.2 | Tanzania (Mwanza), 2012-13(22) | Tanzania (Mwanza), 2012-13(22) |
| Timor-Leste | Lower middle income | 27.8 | SEARO region | Asia, Southeast |
| Togo | Low income | 29.2 | AFRO region | Sub-Saharan Africa, West |
| Tonga | Upper middle income | 22.9 | WPRO region, excluding China & Australia | Oceania |
| Turkmenistan | Upper middle income | 22.9 | EURO region | Asia, Central |
| Uganda | Low income | 29.2 | AFRO region | Sub-Saharan Africa, East |
| Ukraine | Lower middle income | 27.8 | EURO region | Europe, Eastern |
| Uzbekistan | Lower middle income | 27.8 | EURO region | Asia, Central |
| Vanuatu | Lower middle income | 27.8 | WPRO region, excluding China & Australia | Oceania |
| Vietnam | Lower middle income | 27.8 | Central Vietnam, 2009-2010(70) | Central Vietnam, 2009-2010(70) |
| Yemen | Lower middle income | 27.8 | Yemen, 1985(45) & 2002-3(46) | Yemen, 1985(45) & 2002-3(46) |
| Zambia | Lower middle income | 27.8 | Zambia, 1979-80 (23) | Zambia, 1979-80 (23) |
| Zimbabwe | Low income | 29.2 | AFRO region | Sub-Saharan Africa, Southern |

Table S.2: Datasets used to set up bootstrap files for the WHO Regions, updated from (1) with the additional datasets identified since then.

| **Region** | **Datasets** |
| --- | --- |
| African (AFRO) | Benin, 1993(2); Burkina Faso, 2007-8(3); Congo, <1991(4); Cote d'Ivoire, 1975(5) & 1985-6(6); Democratic Republic of the Congo (Kikwit, Mikalayi, Tshikapa, Vanga), 2008-9(7); Ethiopia, 1981(8) & 1994(9); Gabon, 1985(10); Ghana, 1997(11); Kenya, 1996-9 (Kilifi)(12, 13) and 2005 (Eldoret) (14); Madagascar, 1990-1995(15); Mozambique, 2002(16); Nigeria, <1978(17), <2002(18) & 2007-8(19); Senegal, 1996-2001(20); South Africa, 2003(21); Tanzania (Mwanza), 2012-13(22); Zambia, 1979-80(23), |
| American, excluding Caribbean (AMRO, excl Caribbean) | Argentina, 1967-8 (urban & rural)(24), & 1981 (Mar de Plata)(25); Brazil, 1967-8(24), 1987(26) & 1996-8(27); Canada, <1967(28); Chile 1967-8 (Santiago & rural)(24); Mexico, 1987-88(29) & 1989(30); Panama 1967-8 (Panama City & rural)(24); Peru, 1967-8 (Lima & rural)(24) & 2003(31); Uruguay, 1967-7 (urban and rural)(24); USA <1967 (Atlanta & Houston)(28). |
| Caribbean | Haiti, 2003(32), Jamaica, 1967-8 (Kingston & rural)(24), Trinidad 1966-7(33), 1967-8 (Port au Spain & rural)(24) |
| Eastern Mediterranean (EMRO) | Bahrain, 1981(34); Iran, 1993-95(35); Jordan, 1982-3(36); Kuwait, <1978(37); Lebanon, 1980-1(38); Morocco, 1969-70(39); Pakistan, <1997(40) & 1999-2004(41); Saudi Arabia, 1989(42) & 1992-93(43), Tunisia, <1970(44); Yemen, 1985(45) & 2002-03(46) |
| European (EURO) | Czech Republic, <1967(28); Denmark, <1967(28) &1983(47); East Germany, 1990(48); England, <1967(28) & 1986-7(49); Finland, 1979(50); France, <1967(28); Kyrgyzstan, 2001(51); Romania, <1989(52); Turkey, 1998(53), 2003-04(54) & 2005(55). |
| South East Asian (SEARO) | Bangladesh, 2004-5(56); India, 1968 (urban & rural Delhi)(57), 1972-3 (Chandrigarh & Lucknow)(57), 1976 (Calcutta)(58), <1987 (Delhi)(59), <1990 (Delhi)(60), 1999-2000 (urban and rural Vellore)(61); Indonesia, 2007 (*S Reef, personal communication, March 2015*); Nepal, 2008(62), Thailand, 1978(63) |
| Western Pacific, excluding China & Australia (WPRO, excluding China & Australia) | Cambodia, 2012(64); Fiji, <1973(65); Japan, <1967 (Sapporo &Ohtsu)(28); Malaysia, <1972(66); Singapore, 1975-79(67), Taiwan, 1984(68) & 1984-6(69); Central Vietnam, 2009-2010(70) |

Table S.3: Datasets used to set up bootstrap files for the Global Burden of Disease (GBD) regions, updated from (1) with the additional datasets identified since then.

| **GBD Region** | **Setting from which dataset(s) were collected** |
| --- | --- |
| Sub-Saharan Africa, Central | Congo, <1991(4); Democratic Republic of the Congo (Kikwit, Mikalayi, Tshikapa, Vanga), 2008-9(7); Gabon, 1985(10); |
| Sub-Saharan Africa, East | Ethiopia, 1981(8) & 1994(9); Kenya, 1996-9 (Kilifi)(12, 13) and 2005 (Eldoret) (14); Madagascar, 1990-1995(15); Mozambique, 2002(16); Tanzania (Mwanza), 2012-13(22); Zambia, 1979-80(23) |
| Sub-Saharan Africa, Southern | South Africa, 2003(21) |
| Sub-Saharan Africa, West | Benin, 1993(2); Burkina Faso, 2007-8(3); Cote d'Ivoire, 1975(5) & 1985-6(6); Ghana, 1997(11); Nigeria, <1978(17), <2002(18) & 2007-8(19); Senegal, 1996-2001(20) |
| Caribbean | Haiti, 2003(32), Jamaica, 1967-8 (Kingston & rural)(24), Trinidad 1966-7(33), 1967-8 (Port au Spain & rural)(24) |
| Latin America, Andean | Peru, 1967-8 (Lima & rural)(24) & 2003(31) |
| Latin America, Central | Mexico, 1987-88(29) & 1989(30), Panama 1967-8 (Panama City & rural)(24) |
| Latin America, Southern | Argentina, 1967-8 (urban & rural)(24), & 1981 (Mar de Plata)(25), Chile (Santiago & rural), 1967-8(24); Uruguay, 1967-7 (urban and rural)(24) |
| Latin America, Tropical | Brazil, 1967-8(24), 1987(26) & 1996-8(27) |
| North America, High Income | Canada, <1967(28), USA <1967 (Atlanta & Houston)(28) |
| Asia Central | Kyrgyzstan, 2001(51) |
| North Africa / Middle East | Bahrain, 1981(34); Iran, 1993-95(35); Jordan, 1982-3(36); Kuwait, <1978(37); Lebanon, 1980-81(38); Morocco, 1969-1970(39); Saudi Arabia, 1989(42) & 1992-93(43) Tunisia, <1970(44); Turkey, 1998(71), 2003-4(54) & 2005(55); Yemen, 1985(45) & 2002-03(46) |
| Europe, Eastern | Taken to be identical to those for Europe Central (Romania, <1989(52); Czech Republic, <1967(28)), as no datasets were available from the countries in this grouping |
| Europe Central | Romania, <1989(52); Czech Republic, <1967(28) |
| Europe, Western | Denmark, <1967(28) &1983(47); England, 1986-87(49) & <1967(28); East Germany, 1990(48); Finland, 1979(50); France, <1967(28). |
| Asia East | China, 1979-80(72); Taiwan, 1984(68) & 1984-6(69) |
| Asia, South | Bangladesh 2004-5(56); India, 1968 (urban & rural Delhi)(57), 1972-3 (Chandrigarh & Lucknow)(57), 1976 (Calcutta)(58), <1987 (Delhi)(59), <1990 (Delhi)(60), 1999-2000 (urban & rural Vellore)(61); Nepal, 2008(62), Pakistan, <1997(40) & 1999-2004(41) |
| Asia Pacific, High Income | Japan, <1967 (Ohtsu & Sapporo)(28); Singapore, 1975-9(67) |
| Asia, Southeast | Cambodia, 2012(64); Indonesia, 2007 (*S. Reef, personal communication, March 2015*); Malaysia, <1972(66); Thailand, 1978(63); Central Vietnam, 2009-2010(70) |
| Australasia | Australia, <1967(28) |
| Oceania | Fiji, <1973(65) |

**B. Analyses of additional seroprevalence datasets**

The methods used to estimate the force of infection for the seroprevalence datasets identified since the previous related analyses are described in (1). In brief, four catalytic models (A, B, C, D) were fitted to the age-stratified seroprevelence data to estimate the average annual “force of infection” among <13 and ≥13 year olds (i.e. the rate at which susceptible <13 and ≥13 year olds are infected), and the sensitivity of the antibody assay. The criteria for selecting the force of infection for further use are described in (1), with the added criterion for countries for which the sensitivity of the assay was known to be high that model B was selected in preference to model A if all the other criteria were satisfied and the estimated sensitivity of the assay was 100%for model A, and the lower limit of the 95% confidence interval was implausibly low (less than 95%)(7). Table S.4 summarises the best-fitting values for the force of infection for the additional datasets for which the analyses have not yet been published.

Table S.4: Summary of the additional datasets that were identified since the previous systematic review, best-fitting values for the force of infection and (where appropriate) the sensitivity of the antibody assay, and the CRS incidence per 100,000 live births for each catalytic model before the introduction of RCV. The values in parentheses reflect the 95% confidence intervals, obtained by bootstrapping. To facilitate comparisons, the CRS incidence is not weighted by the number of live births. Analyses for the data from Cambodia are published elsewhere and for brevity are not included here(64).

| **Country, year of study** | **Study population** | **Sample size (no. of age groups)** | **Lab test (cut-off)** | **Cata-lytic model** | **Force of infection (/1000/year)** | | **Sensitivity (%)** | **CRS/ 100,000 live births** | **Loglike-lihood deviance (deg of freedom)** | **Selected model** |
| --- | --- | --- | --- | --- | --- | --- | --- | --- | --- | --- |
|  |  |  |  |  | **<13 yr olds** | **≥13 yr olds** |  |  |  |  |
| Burkina Faso, 2007-8(3) | Pregnant F | 341 (4) | ELISA | A | 0 (0,915) | 828 (0,1000) | 96 (94,100) | 116 (0,220) | 2(1) | B |
|  |  |  |  | B | 242 (135,282) | 3 (0,128) | - | 3 (0,97) | 2(2) |  |
|  |  |  |  | C | 235 (139,990) | 235 (139,990) | 96 (93,99) | 22 (0,89) | 2(2) |  |
|  |  |  |  | D | 126 (108,154) | 126 (108,154) | - | 106 (71,136) | 10(3) |  |
| Democratic Republic of the Congo (Kikwit), 2008-9(7) | Pregnant F | 254 (5) | ELISA, ≥10IU | A | 145 (103,632) | 27 (0,75) | 100 (89,100) | 57 (0,135) | 5(2) | B |
|  |  |  |  | B | 145 (105,189) | 27 (0,69) | - | 57 (0,135) | 5(3) |  |
|  |  |  |  | C | 999 (83,999) | 999 (83,999) | 89 (85,100) | 0 (0,185) | 6(3) |  |
|  |  |  |  | D | 86 (74,103) | 86 (74,103) | - | 179 (145,207) | 10(4) |  |
| Democratic Republic of the Congo (Mikalayi), 2008-9(7) | Pregnant F | 206 (5) | ELISA, ≥10IU | A | 0 (0,466) | 557 (0,992) | 82 (77,100) | 208 (0,331) | 0(2) | B |
|  |  |  |  | B | 103 (66,138) | 23 (0,68) | - | 87 (0,219) | 1(3) |  |
|  |  |  |  | C | 125 (67,969) | 125 (67,969) | 84 (76,99) | 108 (0,220) | 1(3) |  |
|  |  |  |  | D | 63 (54,77) | 63 (54,77) | - | 229 (198,248) | 6(4) |  |
| Democratic Republic of the Congo (Tshikapa), 2008-9 (7) | Pregnant F | 182 (5) | ELISA, ≥10IU | A | 128 (75,913) | 20 (0,248) | 100 (82,100) | 58 (0,187) | 4(2) | B |
|  |  |  |  | B | 128 (84,169) | 20 (0,73) | - | 58 (0,180) | 4(3) |  |
|  |  |  |  | C | 168 (77,999) | 168 (77,999) | 86 (80,100) | 58 (0,200) | 4(3) |  |
|  |  |  |  | D | 76 (62,94) | 76 (62,94) | - | 202 (163,232) | 9(4) |  |

Table S.4 continued

| **Country, year of study** | **Study population** | **Sample size (no. of age groups)** | **Lab test (cut-off)** | **Cata-lytic model** | **Force of infection (/1000/year)** | | **Sensitivity (%)** | **CRS/ 100,000 live births** | **Loglike-lihood deviance (deg of freedom)** | **Selected model** |
| --- | --- | --- | --- | --- | --- | --- | --- | --- | --- | --- |
|  |  |  |  |  | **<13 yr olds** | **≥13 yr olds** |  |  |  |  |
| Democratic Republic of the Congo (Vanga), 2008-9 (7), | Pregnant F | 255 (5) | ELISA, ≥10IU | A | 132 (90,252) | 32 (0,75) | 100 (91,100) | 75 (0,165) | 5(2) | B |
|  |  |  |  | B | 132 (91,178) | 32 (0,72) | - | 75 (0,164) | 5(3) |  |
|  |  |  |  | C | 968 (77,999) | 968 (77,999) | 87 (84,100) | 0 (0,200) | 7(3) |  |
|  |  |  |  | D | 83 (71,97) | 83 (71,97) | - | 187 (157,211) | 9(4) |  |
| Indonesia, 2007 (*S Reef, personal communication, March 2015*) | General population | 11320 (10) | ? | A | 135 (119,148) | 62 (32,97) | 93 (91,97) | 93 (68,118) | 3(7) | B |
|  |  |  |  | B | 127 (120,135) | 22 (18,26) | - | 64 (51,77) | 12(8) |  |
|  |  |  |  | C | 115 (106,126) | 115 (106,126) | 91 (90,92) | 124 (106,140) | 12(8) |  |
|  |  |  |  | D | 61 (60,63) | 61 (60,63) | - | 234 (230,237) | 462(9) |  |
| Kenya (Eldoret), 2005(14) | Pregnant F | 437(4) | EIA, ≥10IU | A | 140 (0,219) | 147 (24,875) | 97 (93,100) | 86 (24,239) | 0(1) | B |
|  |  |  |  | B | 154 (91,223) | 66 (1,152) | - | 75 (1,163) | 1(2) |  |
|  |  |  |  | C | 142 (107,950) | 142 (107,950) | 97 (93,100) | 85 (0,137) | 0(2) |  |
|  |  |  |  | D | 113 (100,131) | 113 (100,131) | - | 127 (99,151) | 2(3) |  |
| Tanzania (Mwanza), 2012-13(22) | Pregnant F | 342 (3) | EIA, ≥10IU | A | 0 (0,226) | 544 (20,760) | 96 (93,100) | 214 (19,280) | 0(0) | B |
|  |  |  |  | B | 142 (74,216) | 79 (6,190) | - | 88 (9,181) | 1(1) |  |
|  |  |  |  | C | 135 (105,265) | 135 (105,265) | 98 (93,100) | 93 (14,141) | 0(1) |  |
|  |  |  |  | D | 115 (99,137) | 115 (99,137) | - | 123 (91,153) | 1(2) |  |

## C. Expression for the number of CRS cases.

Country-specific numbers of CRS cases in year *y* during 2001-2080 were calculated by summing the number of CRS cases born each day to women aged 15-44 years, as follows:

$$\sum_{t=1}^{365} \sum_{a=15}^{44} \frac{0.65s_{w}(a,t)f\left( a,y \right)N_{w}\left( a,y \right)(1-e^{-{112\lambda}_{o}\left( t \right)})}{365}$$

*s_w_(a,t)* is the modelled proportion of women aged *a* on day *t* that are susceptible, *f(a,y)* and *N_w_(a,y)* are the fertility rate and population size respectively among women aged *a* in year *y* in the UN population data, and *λ_o_(t)* is the daily model-generated force of infection among women on day *t*.

Figure S.1: Predicted annual number of CRS cases since 2000, if SIA or routine RCV vaccination coverage is at best-estimate level throughout 2001-30 (black line) except for the stated vaccination period of interest, when it is zero or at levels likely to be seen without additional support (red line) (“best-estimate outside, reduced inside”).

Figure S.2: Predicted cumulative number of CRS cases since the start of each period of interest, if SIA or routine RCV vaccination coverage is at best-estimate level throughout 2001-30 (black line) except for the stated vaccination period of interest, when it is at zero or at levels likely to be seen without additional support (red line) (“best-estimate outside, reduced inside”).

Figure S.3: Predicted annual number of CRS cases since 2000, if SIA or RCV vaccination coverage are either at zero or at levels likely to be seen without additional support throughout 2001-30 (black line), except for the stated vaccination period of interest (red line), when they are at best-estimate levels (“reduced outside, best-estimate within”).

Figure S.4: Predicted cumulative number of CRS cases since the start of each vaccination period of interest, if SIA or RCV vaccination coverage are either at zero or at levels likely to be seen without additional support throughout 2001-30 (black line), except for the stated period of interest, when they are at best-estimate levels (red line) (“reduced outside, best-estimate within”).

**References**

1. Vynnycky E, Adams EJ, Cutts FT, Reef SE, Navar AM, Simons E, et al. Using Seroprevalence and Immunisation Coverage Data to Estimate the Global Burden of Congenital Rubella Syndrome, 1996-2010: A Systematic Review. PLoS One. 2016;11(3):e0149160.

2. Rodier MH, Berthonneau J, Bourgoin A, Giraudeau G, Agius G, Burucoa C, et al. Seroprevalences of Toxoplasma, malaria, rubella, cytomegalovirus, HIV and treponemal infections among pregnant women in Cotonou, Republic of Benin. Acta tropica. 1995;59(4):271-7.

3. Tahita MC, Hubschen JM, Tarnagda Z, Ernest D, Charpentier E, Kremer JR, et al. Rubella seroprevalence among pregnant women in Burkina Faso. BMC Infect Dis. 2013;13:164.

4. Yala F, Biendo M, Odongo I, Kounkou R. [Virological and bacteriological study of materno-fetal infections in Brazzaville]. Bulletin de la Societe de pathologie exotique (1990). 1991;84(5 Pt 5):627-34.

5. Vrinat M, Dutertre J, Helies H, Ropero P. [A serological survey of rubella among pregnant women in Abidjan (author's transl)]. Medecine tropicale : revue du Corps de sante colonial. 1978;38(1):53-7.

6. Ouattara SA, Brettes JP, Kodjo R, Penali K, Gershy-Damet G, Sangare A, et al. [Seroepidemiology of rubella in the Ivory Coast. Geographic distribution]. Bulletin de la Societe de pathologie exotique et de ses filiales. 1987;80(4):655-64.

7. Alleman MM, Wannemuehler KA, Hao L, Perelygina L, Icenogle JP, Vynnycky E, et al. Estimating the burden of rubella virus infection and congenital rubella syndrome through a rubella immunity assessment among pregnant women in the Democratic Republic of the Congo: Potential impact on vaccination policy. Vaccine. 2016;34(51):6502-11.

8. Sandow D, Okubagzhi GS, Arnold U, Denkmann N. Seroepidemiological study in rubella in pregnant women in Gondar Region, northern Ethiopia. Ethiopian medical journal. 1982;20(4):173-8.

9. Cutts FT, Abebe A, Messele T, Dejene A, Enquselassie F, Nigatu W, et al. Sero-epidemiology of rubella in the urban population of Addis Ababa, Ethiopia. Epidemiol Infect. 2000;124(3):467-79.

10. Mefane C. Rubella antibodies in 1737 girls and women in Gabon. Afrique Medicale. 1985;24(226):29-32.

11. Lawn JE, Reef S, Baffoe-Bonnie B, Adadevoh S, Caul EO, Griffin GE. Unseen blindness, unheard deafness, and unrecorded death and disability: congenital rubella in Kumasi, Ghana. American journal of public health. 2000;90(10):1555-61.

12. Cumberland P, Shulman CE, Maple PA, Bulmer JN, Dorman EK, Kawuondo K, et al. Maternal HIV infection and placental malaria reduce transplacental antibody transfer and tetanus antibody levels in newborns in Kenya. J Infect Dis. 2007;196(4):550-7.

13. Scott S, Cumberland P, Shulman CE, Cousens S, Cohen BJ, Brown DW, et al. Neonatal measles immunity in rural Kenya: the influence of HIV and placental malaria infections on placental transfer of antibodies and levels of antibody in maternal and cord serum samples. J Infect Dis. 2005;191(11):1854-60.

14. Kombich JM, PC; Borus, PK Seroprevalence of Natural Rubella Antibodies among Antenatal Attendees at Moi Teaching and Referral Hospital, Eldoret, Kenya. Journal of Immunological Techniques in Infectious Diseases. 2012;1(1).

15. Dromigny JA, Pecarrere JL, Ollivier G, Leroy F, Zeller HG. [Seroprevalence of rubella in pregnant women at Antananarivo. Study of 853 sera at the Pasteur Institute in Madagascar]. Archives de l'Institut Pasteur de Madagascar. 1996;63(1-2):53-5.

16. Barreto J, Sacramento I, Robertson SE, Langa J, de Gourville E, Wolfson L, et al. Antenatal rubella serosurvey in Maputo, Mozambique. Trop Med Int Health. 2006;11(4):559-64.

17. Odelola HA. Rubella haemagglutination inhibiting antibodies in females of child-bearing age in western Nigeria. Journal of hygiene, epidemiology, microbiology, and immunology. 1978;22(2):190-4.

18. Bukbuk DN, el Nafaty AU, Obed JY. Prevalence of rubella-specific IgG antibody in non-immunized pregnant women in Maiduguri, north eastern Nigeria. Central European journal of public health. 2002;10(1-2):21-3.

19. Amina MD, Oladapo S, Habib S, Adebola O, Bimbo K, Daniel A. Prevalence of rubella IgG antibodies among pregnant women in Zaria, Nigeria. International Health. 2010;2(2):156-9.

20. Dromigny JA, Nabeth P, Perrier Gros Claude JD. Evaluation of the seroprevalence of rubella in the region of Dakar (Senegal). Trop Med Int Health. 2003;8(8):740-3.

21. Corcoran C, Hardie DR. Seroprevalence of rubella antibodies among antenatal patients in the Western Cape. South African medical journal = Suid-Afrikaanse tydskrif vir geneeskunde. 2005;95(9):688-90.

22. Mwambe B, Mirambo MM, Mshana SE, Massinde AN, Kidenya BR, Michael D, et al. Sero-positivity rate of rubella and associated factors among pregnant women attending antenatal care in Mwanza, Tanzania. BMC Pregnancy Childbirth. 2014;14:95.

23. Watts T. Rubella antibodies in a sample of Lusaka mothers. Medical journal of Zambia. 1983;17(4):109-10.

24. Dowdle WR, Ferrera W, De Salles Gomes LF, King D, Kourany M, Madalengoitia J, et al. WHO collaborative study on the sero-epidemiology of rubella in Caribbean and Middle and South American populations in 1968. Bull World Health Organ. 1970;42(3):419-22.

25. Pereira F, Uez O. Rubella antibodies in female applicants for premarital health certificates in Mar del Plata, Argentina. Bull Pan Am Health Organ. 1986;20(2):179-85.

26. Souza VA, Moraes JC, Sumita LM, Camargo MC, Fink MC, Hidalgo NT, et al. Prevalence of rubella antibodies in a non-immunized urban population, Sao Paulo, Brazil. The Division of Immunization, CVE. Revista do Instituto de Medicina Tropical de Sao Paulo. 1994;36(4):373-6.

27. Reiche EM, Morimoto HK, Farias GN, Hisatsugu KR, Geller L, Gomes AC, et al. [Prevalence of American trypanosomiasis, syphilis, toxoplasmosis, rubella, hepatitis B, hepatitis C, human immunodeficiency virus infection, assayed through serological tests among pregnant patients, from 1996 to 1998, at the Regional University Hospital Norte do Parana]. Rev Soc Bras Med Trop. 2000;33(6):519-27.

28. Rawls WE, Melnick JL, Bradstreet CM, Bailey M, Ferris AA, Lehmann NI, et al. WHO collaborative study on the sero-epidemiology of rubella. Bull World Health Organ. 1967;37(1):79-88.

29. Gutierrez Trujillo G, Munoz O, Tapia Conyer R, Bustamante Calvillo ME, Alvarez y Munoz MT, Guiscafre Gallardo JP, et al. [The seroepidemiology of rubella in Mexican women. A national probability survey]. Salud publica de Mexico. 1990;32(6):623-31.

30. Yamamoto L, Mejia E, Lopez RM, Gallardo E, Gomez B. Susceptibility to rubella infection in females at high risk. Immune protection associated to population density. Tropical and geographical medicine. 1995;47(6):235-8.

31. Suarez-Ognio L, Adrianzen A, Ortiz A, Martinez C, Whittembury A, Cabezudo E, et al. A rubella serosurvey in postpartum women in the three regions of Peru. Rev Panam Salud Publica. 2007;22(2):110-7.

32. Desinor OY, Anselme RJ, Laender F, Saint-Louis C, Bien-Aime JE. Seroprevalence of antibodies against rubella virus in pregnant women in Haiti. Rev Panam Salud Publica. 2004;15(3):147-50.

33. Pitts OM, Ravenel JM, Finklea JF. Rubella immunity in Trinidad. American journal of epidemiology. 1969;89(3):271-6.

34. Dutta SR, Atrash HK, Mathew L, Mathew PP, Mahmood RA. Seroepidemiology of rubella in Bahrain. Int J Epidemiol. 1985;14(4):618-23.

35. Modarres S, Modarres S, Oskoii NN. The immunity of children and adult females to rubella virus infection in Tehran. Iranian journal of medical sciences. 1996;21:69-73.

36. El-Khateeb MS, Tarawneh MS, Hijazi S, Kahwaji L. Seroimmunity to rubella virus in Jordanians. Public Health. 1983;97(4):204-7.

37. Hathout H, Al-Nakib W, Lilley H, Abo-Ahmed HS, Nosseir AF. Seroepidemiology of rubella in Kuwait: an alternative vaccination policy. Int J Epidemiol. 1978;7(1):49-53.

38. Bedrossian NK, Matossian R. Is there a rubella problem in Lebanon? Lebanese Medical Journal. 1985;35(1):31-8.

39. Nejmi S. [Immunologic survey of rubella in Moroccan women in the Rabat region (study of antibodies inhibiting hemagglutination in 548 serums)]. Maroc medical. 1972;52(559):420-5.

40. Iqbal A, Bokhari S. Occurrence of rubella antibody IgG in the general population. Mother and Child. 1997;35(1):17-22.

41. Ahmed R, Hashmi K, Ullah SE, Khanum T, Rafia A. Study of Prevalence of Immune Status in Adult Females For Rubella Virus Infection. Pakistan Journal of Biological Sciences. 2006;9(5):816.

42. Hossain A. Seroepidemiology of rubella in Saudi Arabia. Journal of tropical pediatrics. 1989;35(4):169-70.

43. Saeed A, Abu-Shagra S, Al-Rasheed R. Congenital Rubella Syndrome - revisited (letter). Saudi Med J. 1993;14(25-26).

44. Nabli B. [Seroepidemiology of rubella in Tunisia]. Bull World Health Organ. 1970;42(6):891-6.

45. Strauss J, Dobahi SS, Danes L, Kopecky K, Svandova E. Serological survey of rubella in Yemen in 1985. Journal of hygiene, epidemiology, microbiology, and immunology. 1989;33(2):163-7.

46. Sallam TA, Al-Jaufy AY, Al-Shaibany KS, Ghauth AB, Best JM. Prevalence of antibodies to measles and rubella in Sana'a, Yemen. Vaccine. 2006;24(37-39):6304-8.

47. Glikmann G, Petersen I, Mordhorst CH. Prevalence of IgG-antibodies to mumps and measles virus in non-vaccinated children. Dan Med Bull. 1988;35(2):185-7.

48. Edmunds WJ, Gay NJ, Kretzschmar M, Pebody RG, Wachmann H. The pre-vaccination epidemiology of measles, mumps and rubella in Europe: implications for modelling studies. Epidemiol Infect. 2000;125(3):635-50.

49. Morgan-Capner P, Wright J, Miller CL, Miller E. Surveillance of antibody to measles, mumps, and rubella by age. BMJ. 1988;297(6651):770-2.

50. Ukkonen P. Rubella immunity and morbidity: impact of different vaccination programs in Finland 1979-1992. Scand J Infect Dis. 1996;28(1):31-5.

51. Malakmadze N, Zimmerman LA, Uzicanin A, Shteinke L, Caceres VM, Kasymbekova K, et al. Development of a rubella vaccination strategy: contribution of a rubella susceptibility study of women of childbearing age in Kyrgyzstan, 2001. Clinical infectious diseases : an official publication of the Infectious Diseases Society of America. 2004;38(12):1780-3.

52. Dumitrescu R, Mateescu M, Gaicu N, Comanescu D. Evaluation of the anti-rubella immunity levels on a lot of 5,000 sera from women at procreative age, tested by HAI, in Romania. Archives roumaines de pathologie experimentales et de microbiologie. 1989;48(3):253-63.

53. Aksakal FN, Maral I, Cirak MY, Aygun R. Rubella seroprevalence among women of childbearing age residing in a rural region: is there a need for rubella vaccination in Turkey? Jpn J Infect Dis. 2007;60(4):157-60.

54. Pehlivan E, Karaoglu L, Ozen M, Gunes G, Tekerekoglu MS, Genc MF, et al. Rubella seroprevalence in an unvaccinated pregnant population in Malatya, Turkey. Public Health. 2007;121(6):462-8.

55. Sasmaz T, Kurt AO, Ozturk C, Bugdayci R, Oner S. Rubella seroprevalence in women in the reproductive period, Mersin, Turkey. Vaccine. 2007;25(5):912-7.

56. Nessa A, Islam MN, Tabassum S, Munshi SU, Ahmed M, Karim R. Seroprevalence of rubella among urban and rural Bangladeshi women emphasises the need for rubella vaccination of pre-pubertal girls. Indian journal of medical microbiology. 2008;26(1):94-5.

57. Seth P, Manjunath N, Balaya S. Rubella infection: the Indian scene. Rev Infect Dis. 1985;7 Suppl 1:S64-7.

58. Chakravarty MS, Gupta B, Das BC, Mukherjee MK, Mitra AC, Sarkar JK. Seroepidemiological study of rubella in Calcutta. The Indian journal of medical research. 1976;64(1):87-92.

59. Khare S, Banerjee K, Padubidri V, Rai A, Kumari S, Kumari S. Lowered immunity status of rubella virus infection in pregnant women. The Journal of communicable diseases. 1987;19(4):391-5.

60. Khare S, Gupta HL, Banerjee K, Kumari S, Kumari S, Gupta HL. Seroimmunity to rubella virus infection in young adult females in Delhi. The Journal of communicable diseases. 1990;22(4):279-80.

61. Brown DWJ, Cutts FT, Joseph A. An evaluation of complementary epidemiological methods in a defined population in Southern India for estimating the burden of Congenital Rubella Syndrome. 2004.

62. Upreti SR, Thapa K, Pradhan YV, Shakya G, Sapkota YD, Anand A, et al. Developing rubella vaccination policy in Nepal--results from rubella surveillance and seroprevalence and congenital rubella syndrome studies. J Infect Dis. 2011;204 Suppl 1:S433-8.

63. Desudchit P, Chatiyanonda K, Bhamornsathit S. Rubella antibody among Thai women of childbearing age. Southeast Asian J Trop Med Public Health. 1978;9(3):312-6.

64. Mao B, Chheng K, Wannemuehler K, Vynnycky E, Buth S, Soeung SC, et al. Immunity to polio, measles and rubella in women of child-bearing age and estimated congenital rubella syndrome incidence, Cambodia, 2012. Epidemiol Infect. 2014:1-10.

65. Macnamara FN, Mitchell R, Miles JA. A study of immunity to rubella in villages in the Fiji islands using the haemagglutination inhibition test. J Hyg (Lond). 1973;71(4):825-31.

66. Lam SK. The seroepidemiology of rubella in Kuala Lumpur, West Malaysia. Bull World Health Organ. 1972;47(1):127-9.

67. Doraisingham S, Goh KT. The rubella immunity of women of child-bearing age in Singapore. Annals of the Academy of Medicine, Singapore. 1981;10(2):238-41.

68. Black FL. Measles active and passive immunity in a worldwide perspective. Prog Med Virol. 1989;36:1-33.

69. Yuan CF, Ng HT. Seroepidemiologic study of rubella in Taiwan's female population. American journal of public health. 1988;78(10):1366-7.

70. Miyakawa M, Yoshino H, Yoshida LM, Vynnycky E, Motomura H, Tho le H, et al. Seroprevalence of rubella in the cord blood of pregnant women and congenital rubella incidence in Nha Trang, Vietnam. Vaccine. 2014;32(10):1192-8.

71. Aksit S, Timocin A, Turpculu A. Rubella immunity in pregnant Turkish women. International journal of gynaecology and obstetrics: the official organ of the International Federation of Gynaecology and Obstetrics. 1999;66(1):33-4.

72. Wannian S. Rubella in the People's Republic of China. Reviews of Infectious Diseases. 1985;7(Supp 1):S72.
